# Polycomb repressor complex 1 promotes gene silencing through H2AK119 mono-ubiquitination in acinar-to-ductal metaplasia and pancreatic cancer cells

**DOI:** 10.18632/oncotarget.6717

**Published:** 2015-12-22

**Authors:** Simone Benitz, Ivonne Regel, Tobias Reinhard, Anna Popp, Isabell Schäffer, Susanne Raulefs, Bo Kong, Irene Esposito, Christoph W. Michalski, Jörg Kleeff

**Affiliations:** ^1^ Department of Surgery, Technische Universität München, Munich, Germany; ^2^ Department of Surgery, University of Heidelberg, Heidelberg, Germany; ^3^ Institute of Pathology, Heinrich-Heine University, Duesseldorf, Germany; ^4^ The Royal Liverpool and Broadgreen University Hospitals, Liverpool, United Kingdom; ^5^ Department of Surgery, Heinrich-Heine University, Duesseldorf, Germany

**Keywords:** polycomb repressor complex, histone mono-ubiquitination, pancreatic cancer, differentiation gene silencing

## Abstract

Acinar-to-ductal metaplasia (ADM) occurring in cerulein-mediated pancreatitis or in oncogenic Kras-driven pancreatic cancer development is accompanied by extensive changes in the transcriptional program. In this process, acinar cells shut down the expression of acinar specific differentiation genes and re-express genes usually found in embryonic pancreatic progenitor cells. Previous studies have demonstrated that a loss of acinar-specific transcription factors sensitizes the cells towards oncogenic transformation, ultimately resulting in cancer development. However, the mechanism behind the transcriptional silencing of acinar cell fate genes in ADM and pancreatic cancer is largely unknown. Here, we analyzed whether elevated levels of the polycomb repressor complex 1 (PRC1) components Bmi1 and Ring1b and their catalyzed histone modification H2AK119ub in ADMs and tumor cells, are responsible for the mediation of acinar gene silencing. Therefore, we performed chromatin-immunoprecipitation in *in vitro* generated ADMs and isolated murine tumor cells against the repressive histone modifications H3K27me3 and H2AK119ub. We established that the acinar transcription factor complex Ptf1-L is epigenetically silenced in ADMs as well as in pancreatic tumor cells. For the first time, this work presents a possible mechanism of acinar gene silencing, which is an important prerequisite in the initiation and maintenance of a dedifferentiated cell state in ADMs and tumor cells.

## INTRODUCTION

The exocrine compartment of the pancreas mainly consists of acinar cells producing digestive enzymes. Accumulating evidence indicates, that a certain degree of acinar cell plasticity is necessary in the pathogenesis of pancreatitis as well as in the development of pancreatic ductal adenocarcinoma (PDAC) [1]. Mouse model studies have shown that acute pancreatitis initiates a transient regeneration program, in which acinar cells lose their characteristic phenotype and undergo acinar-to-ductal metaplasia (ADM). Importantly, metaplastic acini are highly susceptible to oncogenic transformation and are thought to be a prerequisite for PDAC development. The activation of oncogenic Kras at physiological levels in adult mice demonstrates, that Kras recapitulates its full oncogenic potential only in pancreatitis-driven ADM cells [2]. Within this process, ADM cells shut down the expression of acinar specific differentiation genes and acquire duct- and progenitor-like cell features [3]. Acinar cell fate is mainly controlled by the pancreas specific transcription factor-1 (PTF1), containing the DNA-binding subunit Ptf1a and either Rbpj (recombining binding protein suppressor of hairless) in pancreas progenitor cells or Rbpjl (Rbpj-like) in differentiated acinar cells [4]. Additionally, the transcription factor Gata6 was identified to regulate acinar cell maintenance in the healthy organ due to its binding on the *Rbpjl* promoter [5]. A loss of acinar determinants, such as *Gata6* or *Ptf1a* favors pancreatic carcinogenesis [6, 7]. Thus, a repression of acinar specific differentiation genes is an essential step in ADM and cancer progression and might be initiated by epigenetic changes.

Polycomb group (PcG) proteins are histone modifying transcriptional repressors and are commonly activated in embryonic and adult stem cells, helping to maintain stem cell identity by silencing differentiation genes [8]. PcGs are arranged in two structurally diverse complexes, the Polycomb Repressor Complexes 1 and 2 (PRC1, PRC2). PRC1 contains the two core components BMI1 (B lymphoma Mo-MLV insertion region 1) and the E3 ubiquitin ligase RING1B (RNF2, ring finger protein 2), which catalyze the monoubiquitination of lysine 119 of histone 2A (H2AK119ub). The catalytic subunit of PRC2, EZH2 (Enhancer of Zeste), tri-methylates lysine 27 of histone 3 (H3K27me3) [9]. Both histone marks are associated with transcriptional silencing. During cell differentiation, the expression of PRC components diminishes and tissue specific genes are expressed through decreasing levels of the repressive histone marks and increasing levels of the activating histone mark H3K4me3 [8]. Considering that elevated expression levels of the PRC components BMI1 and RING1B were detected in a broad spectrum of human tumors [10], we speculate that they might be responsible for the silencing of differentiation genes in tumor cells.

In our study, we hypothesize that during ADM formation acinar-specific differentiation genes are epigenetically silenced through PRC-mediated gene repression, which could ultimately promote pancreatic cancer development. Therefore, we have investigated the functional relevance of Bmi1 and Ring1b re-expression in ADMs and pancreatic cancer cells and identified changes of histone modifications at promoter sites of acinar differentiation genes in the sequence of pancreatic carcinogenesis.

## RESULTS

### High expression of PRC1 members and enrichment of histone mark H2AK119ub in pancreatitis and PDAC mouse models

**Figure 1:**
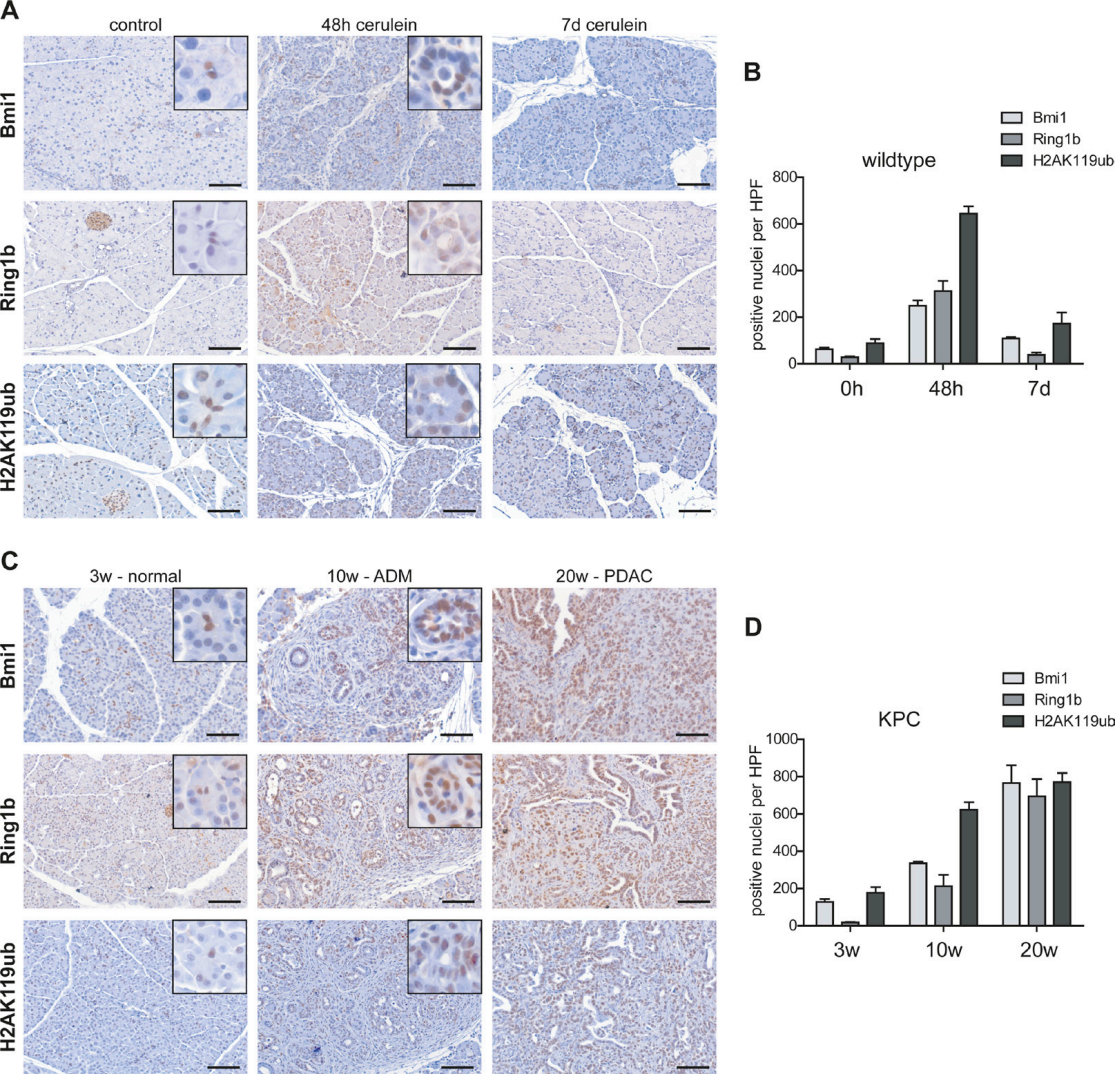
Epigenetic remodelers were overexpressed in pancreatitis and pancreatic cancer (**A**) Immunohistochemistry staining of Bmi1, Ring1b and H2AK119ub in eight week old control mice, 48 hours and seven days after the last cerulein injection (*n* = 5). Representative images include boxes with high magnification to demonstrate nuclear staining of centroacinar (control) and ADM (48h) cells. Bar, 100 μm. (**B**) Positive stained nuclei of Bmi1, Ring1b and H2AK119ub per high power field (HPF) were counted and quantified. Data are presented as mean ± SEM from three pictures of each animal. (**C**) Immunohistochemistry staining against Bmi1, Ring1b and H2AK119ub of pancreatic tissue from three week old KPC mice representing morphological normal tissue, from ten week old mice showing preinvasive ductal lesions and from 20 week old KPC mice demonstrating full PDAC (*n* = 5). High magnification images represent nuclear staining of centroacinar cells (3w-normal) or ADMs (10w-ADM). Bar, 100 μm. (**D**) Positive stained nuclei of Bmi1, Ring1b and H2AK119ub per high power field (HPF) were counted and quantified. Data are presented as mean ± SEM from three pictures of each animal.

Pancreatitis-driven organ disruption is accompanied by acinar-to-ductal metaplasia (ADM) where acinar cells lose their differentiated phenotype [3]. Thus, the expression of the histone modifying PRC1 components Bmi1 and Ring1b and the level of the histone modification H2AK119ub were analyzed at two different points in time following cerulein-induced pancreatitis. In pancreatic tissue of untreated eight week old wildtype mice, the expression of Bmi1 and Ring1b and the occurrence of the histone modification H2AK119ub were mainly restricted to centroacinar, ductal and islet cells, although H2AK119ub staining was also prominent in some acinar cells (Figure 1A, control). At 48 hours after the last cerulein injection, the exocrine compartment was mainly replaced by ADM. In ADM cells, the expression of Bmi1 and Ring1b as well as the presence of H2AK119ub was markedly increased (Figure 1A, 48 h cerulein; Figure 1B). Seven days after the last cerulein injection, the pancreatic tissue was almost recovered and the staining of Bmi1 and Ring1b showed a similar distribution as in the healthy organ. However, H2AK114ub was still present in some regenerative acinar cells (Figure 1A, 7d cerulein). Quantification of the staining clearly confirms rising levels of Bmi1, Ring1b and H2AK119ub in ADMs and decreasing numbers of positive stained nuclei at day seven after the last cerulein injection (Figure 1B).

In pancreatic cancer, ADM is assumed to be the initial step of carcinogenesis and tumor cells are maintained in a dedifferentiated phenotype during neoplastic progression [11]. In order to determine the expression of Bmi1 and Ring1b and the presence of H2AK119ub in pancreatic cancer development, we took advantage of Ptf1a^Cre/+^;LSL-Kras^G12D/+^;Trp53^lox/+^ (KPC) mice, which develop pancreatic cancer within a short time frame. At three weeks of age, centroacinar cells showed high levels of Bmi1, Ring1b and H2AK119ub. Importantly, many acinar cells were slightly positive for Bmi1, Ring1b and H2AK119ub, although the morphology of the exocrine tissue was indistinguishable from wildtype mice (Figure 1C, 3w-normal). Ten week old KPC mice harbored numerous preinvasive lesions, such as atypical flat lesions [12], which were strongly stained for Bmi1, Ring1b and H2AK119ub (Figure 1C, 10w-ADM). After twenty weeks the KPC mice had developed PDAC. Here, tumor cells showed high Bmi1 and Ring1b expression and H2AK119ub was strongly enriched (Figure 1C, 20w-PDAC). The quantification of Bmi1, Ring1b and H2AK119ub positively stained nuclei in KPC mice exhibits that tumor cells harboring high levels of these markers were greatly enriched during pancreatic cancer progression (Figure 1D).

In summary, these results show an elevated expression of Bmi1 and Ring1b and high levels of H2AK119ub in progenitor-like ADM cells as well as in PDAC.

### Elevated levels of PRC1 components and H2AK119ub *in vitro*

**Figure 2:**
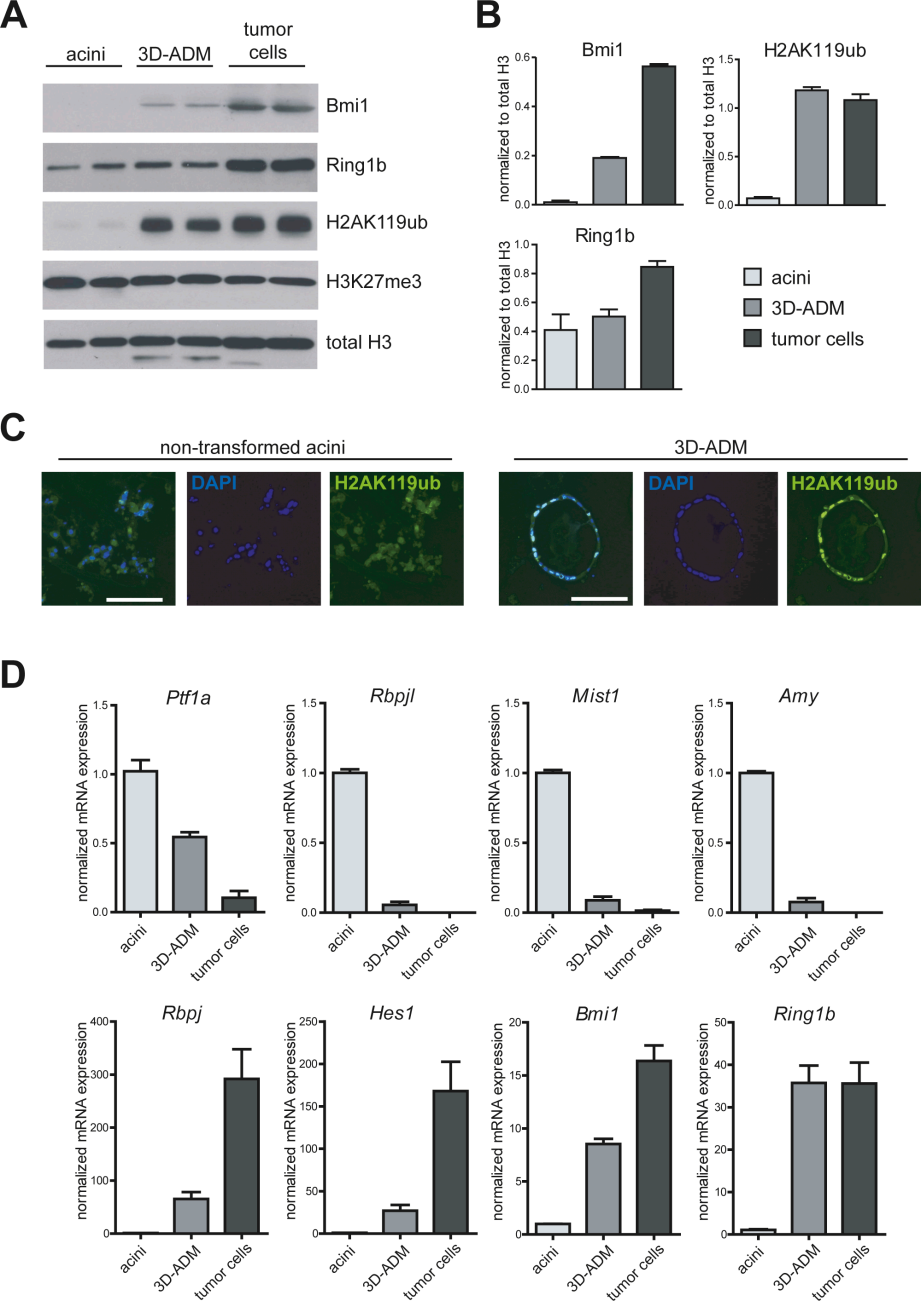
*In vitro* generated sequence of pancreatic carcinogenesis is accompanied by several changes in the transcriptional program (**A**) Protein expression of Bmi1 and Ring1b, as well as total amount of H2AK119ub, H3K27me3 and histone 3 (H3) were analyzed in freshly isolated acinar cells (acini), collagen-embedded acinar cells undergoing ADM (3D-ADM) and previously isolated tumor cells from KC mice with western blot analysis. Two samples of each condition from two independent experiments are shown (*n* = 2). (**B**) Band intensity was measured as mean ± SD and normalized to total H3. (**C**) Representative pictures of H2AK119ub immunofluorescence staining of non-transformed acini and 3D-ADM cells. Nuclei are stained with DAPI. Bar, 100 μm. (**D**) mRNA expression of selected genes was analyzed in acini, 3D-ADM and tumor cells with RT-PCR. Data are normalized to acini and are represented as mean ± SEM (*n* = 3).

To further characterize Bmi1 and Ring1b expression as well as H2AK119ub levels in ADM formation and tumor development, we set up an *in vitro* cell culture system, mimicking the steps of cancer progression from normal acinar cells to ADM and PDAC. For the normal condition we used freshly isolated acinar cells from eight week old wildtype mice. Additionally, we embedded isolated acini of wildtype mice in a 3D-collagen cell culture, where the cells undergo ADM *in vitro* (3D-ADM) and compared both to previously isolated pancreatic tumor cells of a Ptf1a^Cre/+^;LSL-Kras^G12D/+^ (KC) mouse model [13]. In protein analysis, we could detect high levels of Bmi1 in 3D-ADM and tumor cells compared to normal acini, and Ring1b was strongly overexpressed in tumor cells (Figure 2A, 2B). Moreover, H2AK119ub was massively enriched in 3D-ADMs and tumor cells, indicating that PRC1 was catalytically active already in ADM (Figure 2A, 2C). In contrast, the overall amounts of the repressive histone mark H3K27me3, which is catalyzed by PRC2, did not change among normal acini, 3D-ADM and tumor cells (Figure 2A).

It is well described, that ADM is accompanied by alterations in the gene expression program [14, 15]. Thus, we identified a strong repression of selected differentiation genes, such as *Ptf1a, Rbpjl, Mist1* (Bhlha15, basic helix-loop-helix family, member a15) and *Amy* (alpha-Amylase) in 3D-ADMs and pancreatic tumor cells. In contrast, progenitor genes, such as *Rbpj* and *Hes1* (hes family bHLH transcription factor 1) as well as *Bmi1* and *Ring1b* were re-expressed in 3D-ADMs and tumor cells (Figure 2D).

This data indicates that a reactivation of Bmi1 and Ring1b could be responsible for an epigenetic repression of important lineage specific differentiation genes during ADM formation, which become persistently silenced in tumor cells.

### Epigenetic regulation of differentiation and progenitor genes

**Figure 3:**
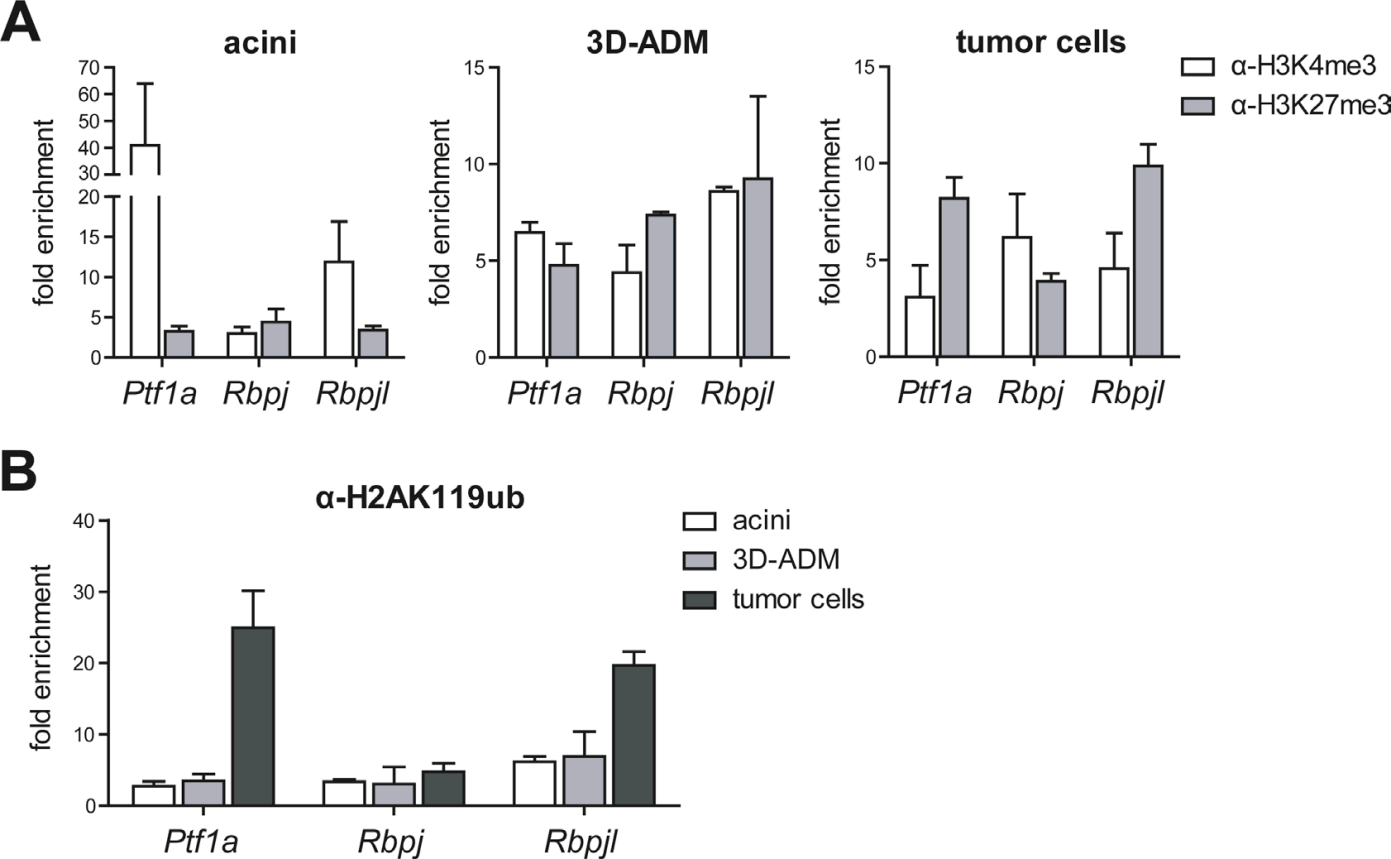
Histone modifications were changed in metaplastic acini and pancreatic tumor cells (**A**) Chromatin-immunoprecipitation (ChIP) followed by RT-PCR was performed in acini, 3D-ADMs and tumor cells with antibodies against H3K4me3 and H3K27me3 and the analysis of the *Ptf1a, Rbpj* and *Rbpjl* promoter. (**B**) Direct comparison of H2AK119ub enrichment in acini, 3D-ADMs and tumor cells was assessed by ChIP-RT-PCR. All ChIP-RT-PCR data are calculated as fold enrichment over IgG control and are represented as mean ± SEM (*n* = 3–5).

High levels of the PRC1 components Bmi1 and Ring1b and the strong repression of differentiation genes in 3D-ADMs and cancer cells let us suppose that PRC1 is directly involved in acinar gene regulation. Therefore, we determined the epigenetic profile of *Ptf1a, Rbpj* and *Rbpjl*, which are the main transcriptional regulators controlling acinar cell fate [4]. From our data we observed that in normal acini the promoter of the embryo-specific transcription factor *Rbpj* showed a slight enrichment of the repressive histone mark H3K27me3. In contrast, *Ptf1a* and *Rbpjl*, which are expressed in differentiated acinar cells, possessed high levels of the activating histone modification H3K4me3 at their promoter sites. This distribution was clearly changed during carcinogenesis. ADM cells showed a bivalent state with similar levels of H3K4me3 and H3K27me3, whereas tumor cells exhibited an enrichment of H3K27me3 at the *Ptf1a* and *Rbpjl* promoter and a slightly elevated H3K4me3 modification at the *Rbpj* promoter (Figure 3A). Importantly, the analysis of the repressive histone modification H2AK119ub revealed that the *Ptf1a* and *Rbpjl* promoter displayed a strong enrichment of H2AK119ub in tumor cells, indicating that acinar specific differentiation genes are persistently silenced during tumor formation. In contrast, H2AK119ub levels did not sequentially change at the *Rbpj* promoter (Figure 3B). The distribution of the epigenetic modifications perfectly matches the gene expression data showing a reactivation of the progenitor gene *Rbpj* and a repression of *Ptf1a* and *Rbpjl* in 3D-ADM and tumor cells.

### Gata6 in the *in vitro* sequence of pancreatic carcinogenesis

**Figure 4:**
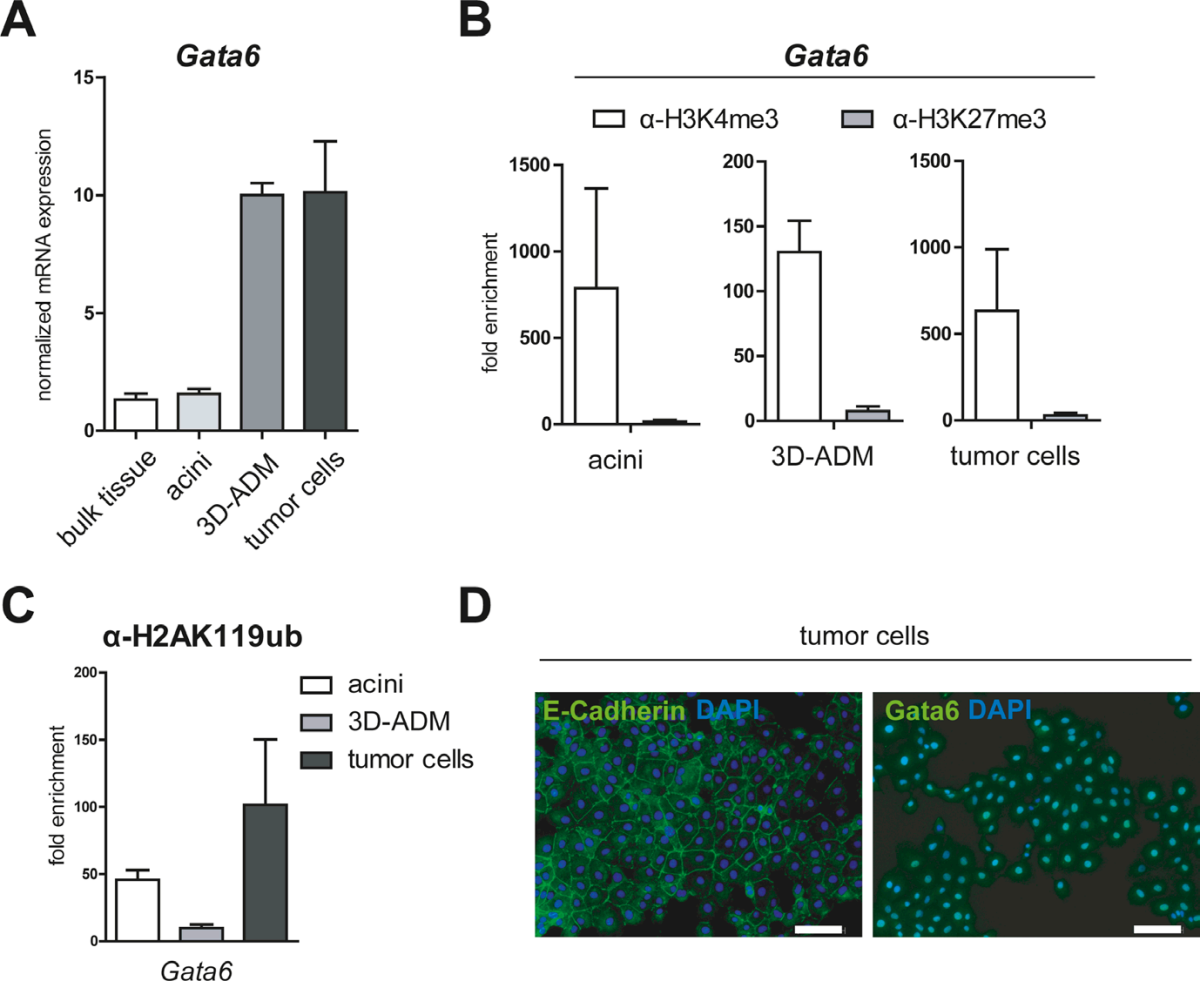
Gata6 is persistently activated in the *in vitro* sequence of pancreatic carcinogenesis (**A**) mRNA expression of *Gata6* was analyzed in bulk pancreatic tissue, acini, 3D-ADM and tumor cells with RT-PCR. Data are normalized to bulk tissue and are represented as mean ± SEM (*n* = 3). (**B**, **C**) Epigenetic pattern of the *Gata6* promoter was examined with ChIP-RT-PCR in acini, 3D-ADMs and tumor cells with antibodies against H3K4me3, H3K27me3 and (C) H2AK119ub. All ChIP-RT-PCR data are calculated as fold enrichment over IgG control and are represented as mean ± SEM (*n* = 3–5). (**D**) Representative pictures of E-Cadherin and Gata6 immunofluorescence staining of tumor cells. Nuclei are stained with DAPI. Bar, 100 μm.

Referring to the study of Martinelli et al., Gata6 was depicted as an important mediator of acinar cell maintenance regulating among others the acinar specific differentiation gene *Rbpjl* [5]. Therefore, we examined the expression of *Gata6* in our *in vitro* pancreatic carcinogenesis model and found unexpectedly an increased gene expression of the acinar transcription factor in 3D-ADMs and in tumor cells. To exclude an induced downregulation of *Gata6* caused by the acinar isolation process, we additionally compared the *Gata6* gene expression to bulk pancreatic tissue and found equal expression levels (Figure 4A). The determination of epigenetic modifications of the *Gata6* promoter in acinar cells, 3D-ADMs and tumor cells showed a persistent and strong enrichment of the active histone mark H3K4me3 (Figure 4B). In addition, the level of the PRC1-catalyzed histone modification H2AK119ub on the *Gata6* promoter demonstrated no major changes (Figure 4C). Here again, the identified epigenetic patterns corresponded perfectly to the gene expression data. However, Gata6 was described to have tumor suppressor capacities and an endogenous downregulation was associated with late and poorly differentiated tumor stages [6]. Therefore, we stained our tumor cells for Gata6 and E-Cadherin, a marker for epithelial differentiation, and detected high levels of membranous E-Cadherin as well as high levels of Gata6 localized in the nuclei (Figure 4D). Consequently, the data demonstrate that our tumor cells are well differentiated in which Gata6 remains expressed.

### Epigenetic regulation of the PRC1 complex member Bmi1

**Figure 5:**
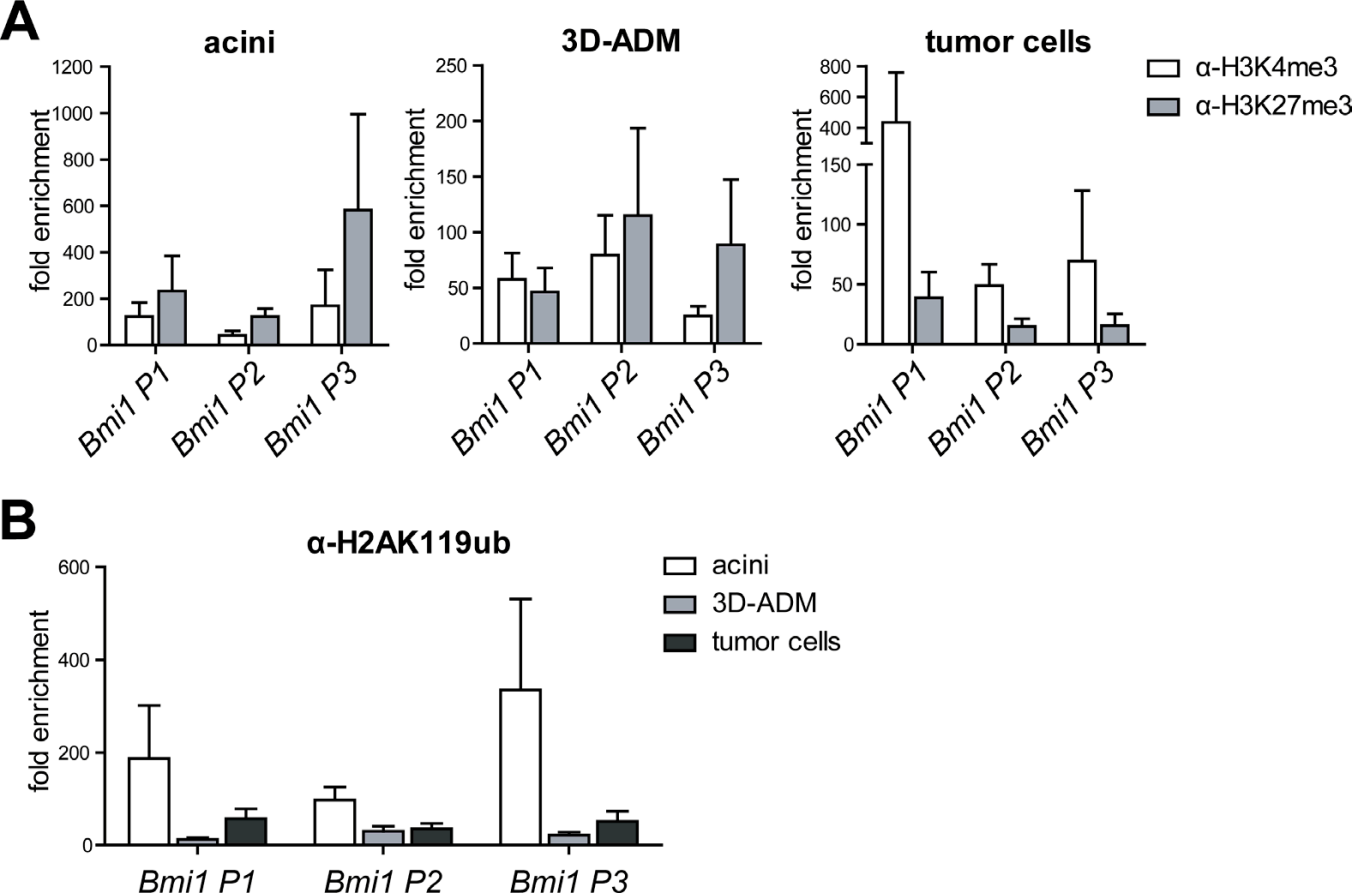
Histone modifications on the Bmi1 promoter were changed in the *in vitro* sequence of pancreatic carcinogenesis (**A**) Epigenetic signature of three *Bmi1* promoter regions (P1 to P3 in the direction from 5′ to 3′) analyzed with ChIP-RT-PCR using H3K4me3 and H3K27me3 antibody represented in three panels for acini, 3D-ADM and tumor cells. (**B**) Direct comparison of the H2AK119ub loss on the *Bmi1* promoter regions in the *in vitro* sequence of acini, 3D-ADM and tumor cells. All ChIP-RT-PCR data are calculated as fold enrichment over IgG control and are represented as mean ± SEM (*n* = 3–5).

Since Bmi1 was re-activated during pancreatic carcinogenesis, we investigated whether its promoter was also epigenetically regulated. Thus, we performed ChIP analysis against different *Bmi1* promoter sites (P1 to P3 from 5′ to 3′). In comparison to acinar and 3D-ADM cells, tumor cells displayed a clear loss of H3K27me3 and a strong enrichment of H3K4me3 (Figure 5A). Importantly, reduced levels of H2AK119ub were already detectable in 3D-ADM cells as well as in tumor cells (Figure 5B). Thus, a transient *Bmi1* expression as it occurs in ADM cells might be solely achieved through a loss of H2AK119ub, whereas a persistent expression in tumor cells might be additionally guaranteed by reduced H3K27me3 levels.

## DISCUSSION

Nearly a decade ago it was shown, that after cerulein-mediated exocrine cell damage, surviving acinar cells initiate a regeneration program. Thereby, acinar cells lose their characteristic phenotype and re-express genes, which are normally active in embryonic pancreatic progenitor cells [3]. A similar metaplastic transformation of acinar cells was observed in early stages of oncogenic Kras-driven pancreatic cancer mouse models [2]. Transcriptional changes were extensively studied in both conditions and revealed that a loss of acinar specific transcription factors, such as Gata6, Mist1 and Ptf1a, supports ADM formation and, in presence of mutant Kras, tumor development [6, 7, 16]. It was shown that the genetic deletion of Ptf1a in an adult stage lead to a downregulation of acinar fate genes and consequently to extensive ADM formation. The loss of Ptf1a additionally potentiated Kras-mediated transformation and supported the development of malignant lesions [7]. Gata6 was identified as another important transcription regulator of acinar cell fate. In physiological conditions, it mediates acinar cell maintenance, whereas a loss of Gata6 in pancreatic carcinogenesis was associated with late and poorly differentiated tumor stages [5, 6]. However, the mechanisms behind the repression of pancreas specific transcription factors, which favor acinar cell reprogramming in pancreatic diseases are largely unknown. Thus, our study provides a new mechanism for how the acinar cell differentiation genes, *Ptf1a* and *Rbpjl*, which are the main mediators of acinar cell fate, are silenced in ADMs and are kept in a repressive state during carcinogenesis. Here, we took advantage of *in vitro* generated ADMs and isolated tumor cells, which show, similarly to *in vivo* models, a loss of acinar differentiation gene expression [17] as well as elevated levels of the transcriptional repressors Bmi1 and Ring1b and their catalyzed histone modification H2AK119ub. Interestingly, our *in vivo* results show that acinar cells of three week old KPC mice express high levels of Bmi1, Ring1b and H2AK119ub indicating that the cells are retained in an immature epigenetic state, although the cells look morphologically fully differentiated. Previous studies have assigned Bmi1 and Ring1b an important role in acinar cell proliferation and formation of pre-neoplastic lesions, however without emphasizing target genes [18, 19]. We additionally assessed the presence of the repressive histone mark H3K27me3, because it was described that elevated levels of EZH2 and a global loss of H3K27me3 are associated with a poor PDAC prognosis [20]. However, in our *in vitro* sequence of pancreatic carcinogenesis we did not detect overall changes of H3K27me3, which does not exclude dynamic changes of this mark at specific promoter regions.

In fact, we identified alterations of activating and repressive histone modifications on acinar cell transcription factors in acinar cells, ADMs and tumor cells. In agreement with the gene expression data, we detected an enrichment of the repressive histone marks H2AK119ub and H3K27me3 at the *Ptf1a* and *Rbpjl* promoter in tumor cells. ADM cells showed a bivalent stage of H3K4me3 and H3K27me3 on the *Ptf1a*, *Rbpj* and *Rbpjl* promoter, which might explain their plasticity towards acinar cell redifferentiation in pancreatitis-driven regeneration or towards tumor cell dedifferentiation in the presence of oncogenic Kras. We speculate that the bivalent epigenetic state can go in either direction, a loss of the repressive histone marks on differentiation genes can induce acinar redifferentiation, whereas an increase supports persistent gene silencing and the formation of full tumor cells [17]. Our findings, that the acinar specific complex PTF1 is epigenetically silenced in ADM and tumor cells corroborate existing studies showing that a loss of Ptf1a supports acinar reprogramming, which in turn makes the cells more susceptible towards oncogenic transformation [7]. Since it was described that the transcription factor Gata6 regulates the acinar gene *Rbpjl*, we analyzed the *Gata6* gene expression and its epigenetic pattern in our *in vitro* carcinogenesis sequence [5]. Unexpectedly, we found increased *Gata6* gene expression and high levels of the active histone modification H3K4me3 on the *Gata6* promoter in ADM and tumor cells. From these results, we conclude that the epigenetic-driven downregulation of *Rbpjl* within our carcinogenesis model, which reflects early metaplasia and well-differentiated tumor stages, is independent of a Gata6 loss. In the end, our results are in line with the study of Martinelli et al., which showed that a loss of Gata6 most likely occurs in late tumor stages exhibiting a poorly differentiated phenotype [6]. This encourages our observations that acinar differentiation genes are silenced through epigenetic modifications early during tumor development. At a later stage additional loss of Gata6 might ensures a permanent gene silencing.

Moreover, we identified that Bmi1, a member of the PRC1 complex, is itself epigenetically regulated. In acini and even in ADMs, the Bmi1 promoter exhibited a strong enrichment of repressive H3K27me3, whereas tumor cells showed a massive loss of this histone modification. In contrast, a loss of the repressive histone modification H2AK119ub was already detectable in ADM cells, suggesting that *Bmi1* gene expression might already be activated due to declined H2AK119ub levels, whereas a strong and persistent expression in tumors cells is accompanied by a reduction of H3K27me3.

In conclusion, our work presents a novel and important mechanism for how acinar differentiation genes are epigenetically silenced in ADM formation and tumor development. An epigenetic repression of these genes is initiated and maintained by the repressive histone modifications H3K27me3 and H2AK119ub, catalyzed by PRC2 and PRC1, respectively. In addition to the identification of genetic mutations, it is of major importance to elucidate epigenetic alterations. This will increase the knowledge of pancreatic carcinogenesis and open new fields for therapeutic interventions.

## MATERIALS AND METHODS

### Mouse lines

Ptf1a^Cre/+^;LSL-Kras^G12D/+^;Trp53^lox/+^ (KPC) mice were generated from Ptf1a^Cre/+^ (kindly provided by Roland M. Schmid, TU Munich), LoxP-STOP-LoxP-Kras^G12D/+^ and Trp53flox mice (both, Jackson Laboratories). C57BL/6J wildtype animals were obtained from Charles River Laboratories. Induction of pancreatitis was performed through eight hourly intraperitoneal injections of 100 μg/kg body weight cerulein (Sigma Aldrich) dissolved in 0.9% physiological saline for two consecutive days. All animal experiments were performed according to regulatory standards and approved by the government of Bavaria (118–14).

### Cell culture

Pancreatic cancer cell lines were previously isolated from Ptf1a^Cre/+^;LSL-Kras^G12D/+^ (KC) mice according to standard tumor cell isolation protocols and frozen at passage 2 [13].

### Preparation of acinar explant cultures (3D-cultures)

Acinar cells were isolated from whole pancreatic tissue through collagenase digestion (0.5 mg/ml Collagenase-P, Roche), filtered through 100 μm nylon cell strainer (BD Biosciences) and washed several times with HBSS (Hank›s balanced salt solution, Merck Millipore). Acinar cells were resuspended in Waymouth's MB 752/1 medium (Life Technologies GmbH) supplemented with penicillin G, streptomycin, 10% heat inactivated FCS, 0.1 mg/ml soybean trypsin inhibitor (Sigma Aldrich), 20 μg/ml dexamethasone (Sigma Aldrich), 5 mM HEPES (Life Technologies GmbH) and 0.13% NaHCO3. The cellular suspension was added to neutralized rat tail collagen type I (final concentration 1.5 mg/ml, Corning) and the mixture was solidified at 37°C, 5% CO_2_. After solidification supplemented Waymouth's MB 752/1 medium and 50 ng/ml Tgf-alpha (Life Technologies GmbH) was additionally added. Cells were cultivated at 37°C, 5% CO_2_ for six days. 3D-ADMs were recovered through digestion of the collagen gel with 1 mg/ml collagenase VIII (Sigma Aldrich).

### Immunohistochemistry / Immunfluorescence

Staining was performed according to standard protocols using the following antibodies: Bmi1 (1:100, 05–637, Merck Millipore), Ring1b (1:400, NBP1–49966, Novus Biologicals), E-Cadherin (1:100, 3195, Cell Signaling Technology), Gata6 (1:100, 22600, Abcam) and H2AK119ub (1:400, 8240, Cell Signaling Technology).

### Protein Analysis

Cells were lysed in 50 mM Tris-HCl pH 7.4 2% SDS and 2–20 μg of protein extracts were subjected to Western Blot analysis. Following primary antibodies were used: Bmi1 (1:500), Ring1b (1:500, 5694, Cell Signaling Technology), H2AK119ub (1:5000), H3K27me3 (1:1000, 6002, Abcam) and H3 (1:2000, 9715, Cell Signaling Technology). Band intensity was quantified with the Image Studio Lite software (Licor).

### RNA isolation and quantitative real-time PCR

RNA was isolated using the RNeasy Plus Mini Kit (Qiagen) and was reverse-transcribed to cDNA with the Verso cDNA Synthesis Kit (Thermo Fisher Scientific). RT-PCR was carried out using SYBR Green I Mastermix (Roche) and a LightCycler^®^480 (Roche). Primer sequences are listed in a Supplementary Table. Housekeeping gene *Ppib* was used for relative quantification and gene expression was calculated with the ΔΔCt method.

### Chromatin Immunoprecipitation (ChIP)

The ChIP assay was performed as previously described [21]. Anti-H2AK119ub (9 μg, 8240, Cell Signaling Technology), anti-H3K27me3 (6 μg, 07–449, Merck Millipore) and anti-H3K4me3 (4 μg, 9751, Cell Signaling Technology) were applied for chromatin immunoprecipitation. Chromatin was purified using the QIAquick PCR purification kit (Qiagen). Promotor regions of *Ptf1a*, *Rbpj*, *Rbpjl*, *Gata6* and *Bmi1* were amplified with RT-PCR. The primer sequences are listed in a Supplementary Table. Sample values were calculated as fold enrichment over IgG control.

## SUPPLEMENTARY MATERIALS TABLE



## Abbreviations

ADM: acinar-to-ductal metaplasia
PDAC: pancreatic ductal adenocarcinoma
PRC: polycomb repressor complex
PTF: pancreas specific transcription factor
