# Supplementary material for: Polycomb repressor complex 1 promotes gene silencing through H2AK119 mono-ubiquitination in acinar-to-ductal metaplasia and pancreatic cancer cells
Source: Oncotarget. 2015 Dec 22;7(10):11424–33. doi: 10.18632/oncotarget.6717 (PMC4905483; doi:10.18632/oncotarget.6717)
Supplement: Supplementary file 1 [file oncotarget-07-11424-s001.pdf]

Polycomb repressor complex 1 promotes gene silencing through H2AK119 mono-ubiquitination in acinar-to-ductal metaplasia and pancreatic cancer cells

Supplementary Materials

Supplementary Table S1: Primer sequences of genes

qRT-PCR Primer

Gene	forward 5´–3´	reverse 5´–3´
Amylase	TCCACCTGTTTGAGTGGCGCTGGGT	TGGGTGGAGAGACCTGCACACCTGC
Bmi1	GGCTCCAATGAAGACCGAGG	ATCATTCACCTCTTCCTTAGGCT
Hes1	AAAATTCCTCCTCCCCGGTG	TTTGGTTTGTCCGGTGTCG
Mist1	TCCCCAGTTGGAAGGGCCTCA	TCCTGCATGGGTGTTCGGCG
Ptf1a	CTTGCAGGGCACTCTCTTTC	CGATGTGAGCTGTCTCAGGA
Rbpj	GAGGGCGCGTCCCAAAACCC	AAACTTCCCCGCCGATGGAGC
Rbpjl	GTATCGAAGTCAGTGGCGGT	GCAGGCTCAGGTGAGTCAAA
Ring1b	GTTGATTCTCGAGTCTCGCTC	TGAGACATTTCGGCTCCTGC
Gata6	GAAGCGCGTGCCTTCATC	GTAGTGGTTGTGGTGTGACAGTTG
Ppib	GGAGCGCAATATGAAGGTGC	CTTATCGTTGGCCACGGAGG

ChIP Primer

Promoter	forward 5´-3´	reverse 5´-3´
Bmi1 P1	CGGTGACTGTACGTTAGCCT	TCAGCTCATCCCACAGCAAA
Bmi1 P2	TGTGGTGGCAGTTCAAGCTA	CTCCCTAGTGTACTTAGGACCCA
Bmi1 P3	GCCCTTTGGTGGGAACTTGA	TGAAACCACAGGCTCTTTCCT
Ptf1a	GGACCAGATCCGACCCTACT	CCGCTCCTACGTTCTTTGGT
Rbpj	CCCAACCTCGGCACTCAATG	ATGCCACTGATTCCCTACTGG
Rbpjl	GTCGACACCGAATGAACCCA	GATTCGGGCTTCATCCCTCC
Gata6	GCGAGGTAGGGAATACACAAGG	GTAGTGGTTGTGGTGTGACAGTTG
